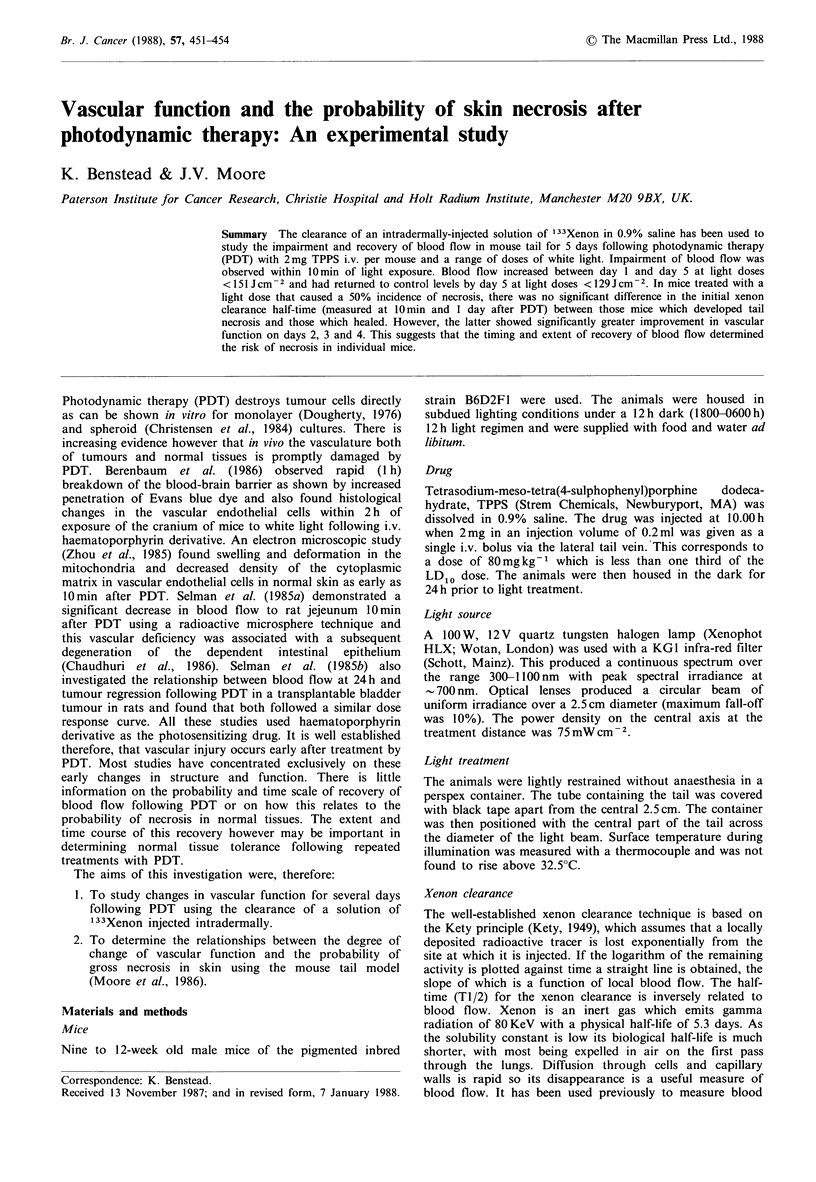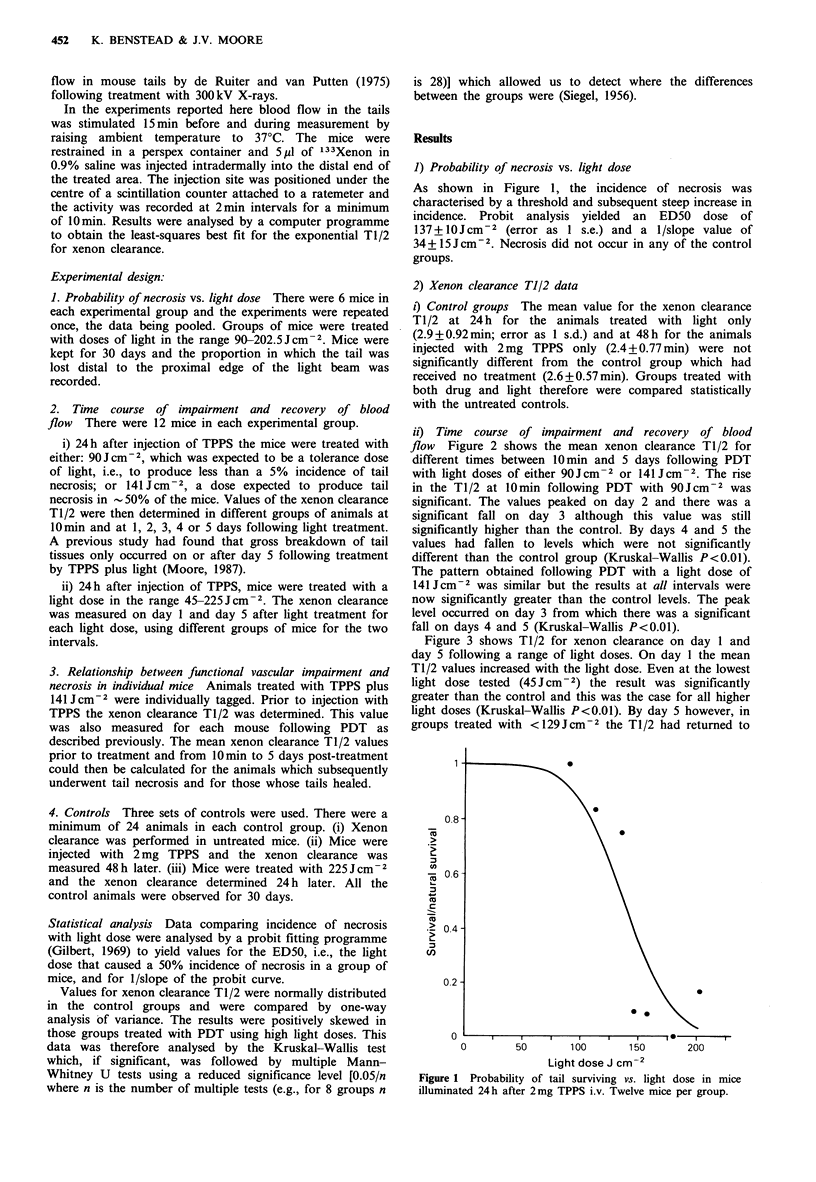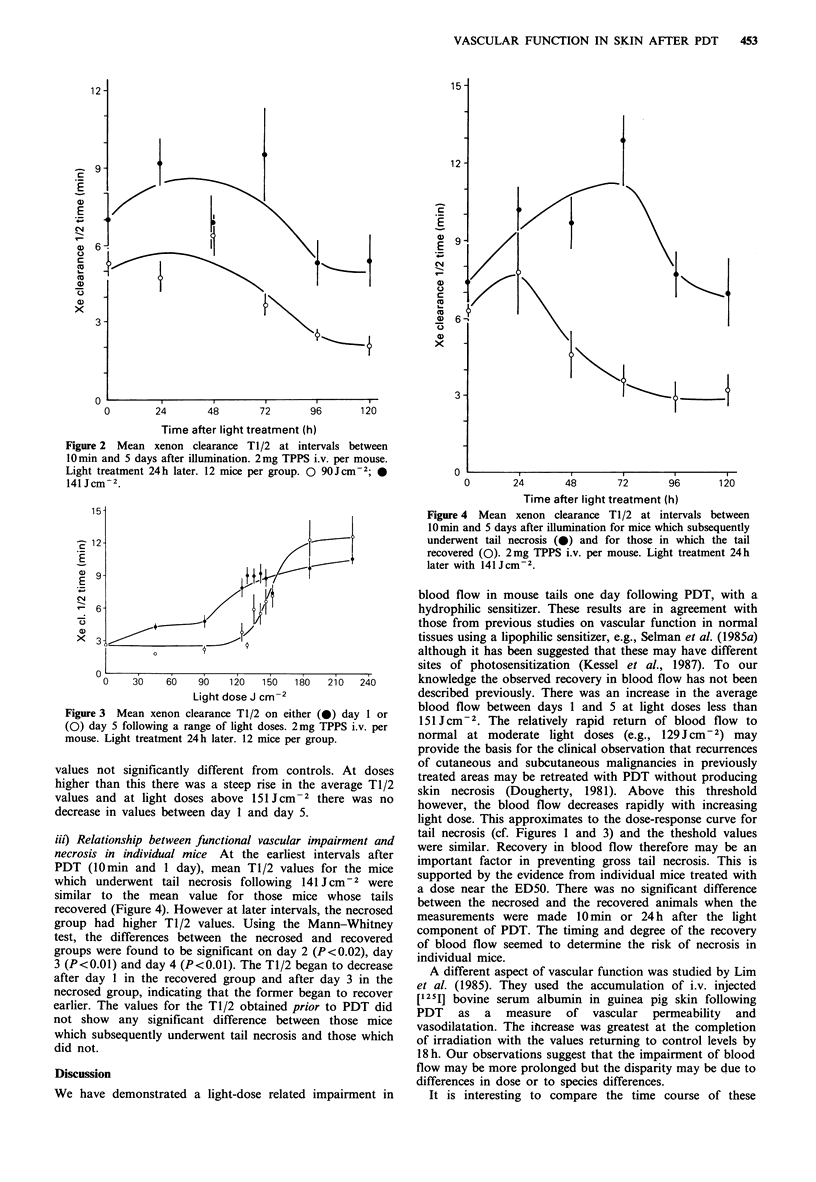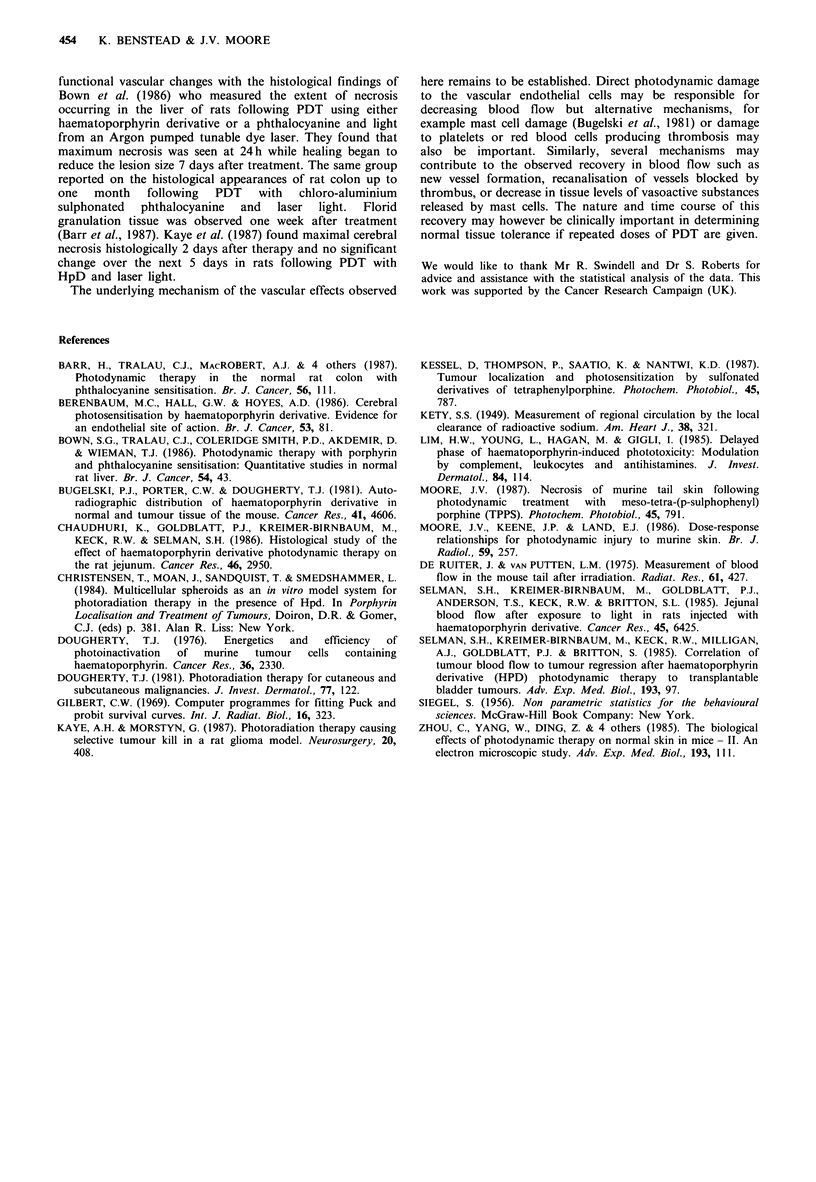# Vascular function and the probability of skin necrosis after photodynamic therapy: an experimental study.

**DOI:** 10.1038/bjc.1988.105

**Published:** 1988-05

**Authors:** K. Benstead, J. V. Moore

**Affiliations:** Paterson Institute for Cancer Research, Christie Hospital and Holt Radium Institute, Manchester, UK.

## Abstract

The clearance of an intradermally-injected solution of 133Xenon in 0.9% saline has been used to study the impairment and recovery of blood flow in mouse tail for 5 days following photodynamic therapy (PDT) with 2mg TPPS i.v. per mouse and a range of doses of white light. Impairment of blood flow was observed within 10 min of light exposure. Blood flow increased between day 1 and day 5 at light doses less than 151J cm-2 and had returned to control levels by day 5 at light doses less than 129J cm-2. In mice treated with a light dose that caused a 50% incidence of necrosis, there was no significant difference in the initial xenon clearance half-time (measured at 10 min and 1 day after PDT) between those mice which developed tail necrosis and those which healed. However, the latter showed significantly greater improvement in vascular function on days 2, 3 and 4. This suggests that the timing and extent of recovery of blood flow determined the risk of necrosis in individual mice.


Br. J. Cancer (1988), 57, 451–454 © The Macmillan Press Ltd., 1988

### Vascular function and the probability of skin necrosis after photodynamic therapy: An experimental study

K. Benstead & J.V. Moore

*Paterson Institute for Cancer Research, Christie Hospital and Holt Radium Institute, Manchester M20 9BX, UK.*

**Summary** The clearance of an intradermally-injected solution of $^{133}$Xenon in 0.9% saline has been used to study the impairment and recovery of blood flow in mouse tail for 5 days following photodynamic therapy (PDT) with 2 mg TPPS i.v. per mouse and a range of doses of white light. Impairment of blood flow was observed within 10 min of light exposure. Blood flow increased between day 1 and day 5 at light doses $<151\,J\,cm^{-2}$ and had returned to control levels by day 5 at light doses $<129\,J\,cm^{-2}$. In mice treated with a light dose that caused a 50% incidence of necrosis, there was no significant difference in the initial xenon clearance half-time (measured at 10 min and 1 day after PDT) between those mice which developed tail necrosis and those which healed. However, the latter showed significantly greater improvement in vascular function on days 2, 3 and 4. This suggests that the timing and extent of recovery of blood flow determined the risk of necrosis in individual mice.

Photodynamic therapy (PDT) destroys tumour cells directly as can be shown *in vitro* for monolayer (Dougherty, 1976) and spheroid (Christensen *et al.*, 1984) cultures. There is increasing evidence however that *in vivo* the vasculature both of tumours and normal tissues is promptly damaged by PDT. Berenbaum *et al.* (1986) observed rapid (1 h) breakdown of the blood-brain barrier as shown by increased penetration of Evans blue dye and also found histological changes in the vascular endothelial cells within 2 h of exposure of the cranium of mice to white light following i.v. haematoporphyrin derivative. An electron microscopic study (Zhou *et al.*, 1985) found swelling and deformation in the mitochondria and decreased density of the cytoplasmic matrix in vascular endothelial cells in normal skin as early as 10 min after PDT. Selman *et al.* (1985*a*) demonstrated a significant decrease in blood flow to rat jejeunum 10 min after PDT using a radioactive microsphere technique and this vascular deficiency was associated with a subsequent degeneration of the dependent intestinal epithelium (Chaudhuri *et al.*, 1986). Selman *et al.* (1985*b*) also investigated the relationship between blood flow at 24 h and tumour regression following PDT in a transplantable bladder tumour in rats and found that both followed a similar dose response curve. All these studies used haematoporphyrin derivative as the photosensitizing drug. It is well established therefore, that vascular injury occurs early after treatment by PDT. Most studies have concentrated exclusively on these early changes in structure and function. There is little information on the probability and time scale of recovery of blood flow following PDT or on how this relates to the probability of necrosis in normal tissues. The extent and time course of this recovery however may be important in determining normal tissue tolerance following repeated treatments with PDT.

The aims of this investigation were, therefore:

1. To study changes in vascular function for several days following PDT using the clearance of a solution of $^{133}$Xenon injected intradermally.
2. To determine the relationships between the degree of change of vascular function and the probability of gross necrosis in skin using the mouse tail model (Moore *et al.*, 1986).

### Materials and methods

### *Mice*

Nine to 12-week old male mice of the pigmented inbred strain B6D2F1 were used. The animals were housed in subdued lighting conditions under a 12 h dark (1800–0600 h) 12 h light regimen and were supplied with food and water *ad libitum.*

### *Drug*

Tetrasodium-meso-tetra(4-sulphophenyl)porphine dodecahydrate, TPPS (Strem Chemicals, Newburyport, MA) was dissolved in 0.9% saline. The drug was injected at 10.00 h when 2 mg in an injection volume of 0.2 ml was given as a single i.v. bolus via the lateral tail vein. This corresponds to a dose of $80\,mg\,kg^{-1}$ which is less than one third of the $LD_{10}$ dose. The animals were then housed in the dark for 24 h prior to light treatment.

### *Light source*

A 100 W, 12 V quartz tungsten halogen lamp (Xenophot HLX; Wotan, London) was used with a KG1 infra-red filter (Schott, Mainz). This produced a continuous spectrum over the range 300–1100 nm with peak spectral irradiance at ~700 nm. Optical lenses produced a circular beam of uniform irradiance over a 2.5 cm diameter (maximum fall-off was 10%). The power density on the central axis at the treatment distance was $75\,mW\,cm^{-2}$.

### *Light treatment*

The animals were lightly restrained without anaesthesia in a perspex container. The tube containing the tail was covered with black tape apart from the central 2.5 cm. The container was then positioned with the central part of the tail across the diameter of the light beam. Surface temperature during illumination was measured with a thermocouple and was not found to rise above 32.5°C.

### *Xenon clearance*

The well-established xenon clearance technique is based on the Kety principle (Kety, 1949), which assumes that a locally deposited radioactive tracer is lost exponentially from the site at which it is injected. If the logarithm of the remaining activity is plotted against time a straight line is obtained, the slope of which is a function of local blood flow. The half-time (T1/2) for the xenon clearance is inversely related to blood flow. Xenon is an inert gas which emits gamma radiation of 80 KeV with a physical half-life of 5.3 days. As the solubility constant is low its biological half-life is much shorter, with most being expelled in air on the first pass through the lungs. Diffusion through cells and capillary walls is rapid so its disappearance is a useful measure of blood flow. It has been used previously to measure blood

Correspondence: K. Benstead.
Received 13 November 1987; and in revised form, 7 January 1988.

flow in mouse tails by de Ruiter and van Putten (1975) following treatment with 300 kV X-rays.

In the experiments reported here blood flow in the tails was stimulated 15 min before and during measurement by raising ambient temperature to 37°C. The mice were restrained in a perspex container and 5 $\mu$l of $^{133}$Xenon in 0.9% saline was injected intradermally into the distal end of the treated area. The injection site was positioned under the centre of a scintillation counter attached to a ratemeter and the activity was recorded at 2 min intervals for a minimum of 10 min. Results were analysed by a computer programme to obtain the least-squares best fit for the exponential T1/2 for xenon clearance.

*Experimental design:*

*1. Probability of necrosis* vs. *light dose* There were 6 mice in each experimental group and the experiments were repeated once, the data being pooled. Groups of mice were treated with doses of light in the range 90–202.5 J cm$^{-2}$. Mice were kept for 30 days and the proportion in which the tail was lost distal to the proximal edge of the light beam was recorded.

*2. Time course of impairment and recovery of blood flow* There were 12 mice in each experimental group.

i) 24 h after injection of TPPS the mice were treated with either: 90 J cm$^{-2}$, which was expected to be a tolerance dose of light, i.e., to produce less than a 5% incidence of tail necrosis; or 141 J cm$^{-2}$, a dose expected to produce tail necrosis in $\sim$50% of the mice. Values of the xenon clearance T1/2 were then determined in different groups of animals at 10 min and at 1, 2, 3, 4 or 5 days following light treatment. A previous study had found that gross breakdown of tail tissues only occurred on or after day 5 following treatment by TPPS plus light (Moore, 1987).

ii) 24 h after injection of TPPS, mice were treated with a light dose in the range 45–225 J cm$^{-2}$. The xenon clearance was measured on day 1 and day 5 after light treatment for each light dose, using different groups of mice for the two intervals.

*3. Relationship between functional vascular impairment and necrosis in individual mice* Animals treated with TPPS plus 141 J cm$^{-2}$ were individually tagged. Prior to injection with TPPS the xenon clearance T1/2 was determined. This value was also measured for each mouse following PDT as described previously. The mean xenon clearance T1/2 values prior to treatment and from 10 min to 5 days post-treatment could then be calculated for the animals which subsequently underwent tail necrosis and for those whose tails healed.

*4. Controls* Three sets of controls were used. There were a minimum of 24 animals in each control group. (i) Xenon clearance was performed in untreated mice. (ii) Mice were injected with 2 mg TPPS and the xenon clearance was measured 48 h later. (iii) Mice were treated with 225 J cm$^{-2}$ and the xenon clearance determined 24 h later. All the control animals were observed for 30 days.

*Statistical analysis* Data comparing incidence of necrosis with light dose were analysed by a probit fitting programme (Gilbert, 1969) to yield values for the ED50, i.e., the light dose that caused a 50% incidence of necrosis in a group of mice, and for 1/slope of the probit curve.

Values for xenon clearance T1/2 were normally distributed in the control groups and were compared by one-way analysis of variance. The results were positively skewed in those groups treated with PDT using high light doses. This data was therefore analysed by the Kruskal–Wallis test which, if significant, was followed by multiple Mann–Whitney U tests using a reduced significance level [0.05/$n$ where $n$ is the number of multiple tests (e.g., for 8 groups $n$ is 28)] which allowed us to detect where the differences between the groups were (Siegel, 1956).

### Results

*1) Probability of necrosis* vs. *light dose*

As shown in Figure 1, the incidence of necrosis was characterised by a threshold and subsequent steep increase in incidence. Probit analysis yielded an ED50 dose of 137 ± 10 J cm$^{-2}$ (error as 1 s.e.) and a 1/slope value of 34 ± 15 J cm$^{-2}$. Necrosis did not occur in any of the control groups.

*2) Xenon clearance T1/2 data*

*i) Control groups* The mean value for the xenon clearance T1/2 at 24 h for the animals treated with light only (2.9 ± 0.92 min; error as 1 s.d.) and at 48 h for the animals injected with 2 mg TPPS only (2.4 ± 0.77 min) were not significantly different from the control group which had received no treatment (2.6 ± 0.57 min). Groups treated with both drug and light therefore were compared statistically with the untreated controls.

*ii) Time course of impairment and recovery of blood flow* Figure 2 shows the mean xenon clearance T1/2 for different times between 10 min and 5 days following PDT with light doses of either 90 J cm$^{-2}$ or 141 J cm$^{-2}$. The rise in the T1/2 at 10 min following PDT with 90 J cm$^{-2}$ was significant. The values peaked on day 2 and there was a significant fall on day 3 although this value was still significantly higher than the control. By days 4 and 5 the values had fallen to levels which were not significantly different than the control group (Kruskal–Wallis $P < 0.01$). The pattern obtained following PDT with a light dose of 141 J cm$^{-2}$ was similar but the results at *all* intervals were now significantly greater than the control levels. The peak level occurred on day 3 from which there was a significant fall on days 4 and 5 (Kruskal–Wallis $P < 0.01$).

Figure 3 shows T1/2 for xenon clearance on day 1 and day 5 following a range of light doses. On day 1 the mean T1/2 values increased with the light dose. Even at the lowest light dose tested (45 J cm$^{-2}$) the result was significantly greater than the control and this was the case for all higher light doses (Kruskal–Wallis $P < 0.01$). By day 5 however, in groups treated with < 129 J cm$^{-2}$ the T1/2 had returned to

**Figure 1** Probability of tail surviving *vs.* light dose in mice illuminated 24 h after 2 mg TPPS i.v. Twelve mice per group.

**Figure 2** Mean xenon clearance T1/2 at intervals between 10 min and 5 days after illumination. 2 mg TPPS i.v. per mouse. Light treatment 24 h later. 12 mice per group. ○ $90 J cm^{-2}$; ● $141 J cm^{-2}$.

**Figure 3** Mean xenon clearance T1/2 on either (●) day 1 or (○) day 5 following a range of light doses. 2 mg TPPS i.v. per mouse. Light treatment 24 h later. 12 mice per group.

values not significantly different from controls. At doses higher than this there was a steep rise in the average T1/2 values and at light doses above $151 J cm^{-2}$ there was no decrease in values between day 1 and day 5.

*iii) Relationship between functional vascular impairment and necrosis in individual mice* At the earliest intervals after PDT (10 min and 1 day), mean T1/2 values for the mice which underwent tail necrosis following $141 J cm^{-2}$ were similar to the mean value for those mice whose tails recovered (Figure 4). However at later intervals, the necrosed group had higher T1/2 values. Using the Mann–Whitney test, the differences between the necrosed and recovered groups were found to be significant on day 2 ($P<0.02$), day 3 ($P<0.01$) and day 4 ($P<0.01$). The T1/2 began to decrease after day 1 in the recovered group and after day 3 in the necrosed group, indicating that the former began to recover earlier. The values for the T1/2 obtained *prior* to PDT did not show any significant difference between those mice which subsequently underwent tail necrosis and those which did not.

### Discussion

We have demonstrated a light-dose related impairment in

**Figure 4** Mean xenon clearance T1/2 at intervals between 10 min and 5 days after illumination for mice which subsequently underwent tail necrosis (●) and for those in which the tail recovered (○). 2 mg TPPS i.v. per mouse. Light treatment 24 h later with $141 J cm^{-2}$.

blood flow in mouse tails one day following PDT, with a hydrophilic sensitizer. These results are in agreement with those from previous studies on vascular function in normal tissues using a lipophilic sensitizer, e.g., Selman *et al.* (1985*a*) although it has been suggested that these may have different sites of photosensitization (Kessel *et al.*, 1987). To our knowledge the observed recovery in blood flow has not been described previously. There was an increase in the average blood flow between days 1 and 5 at light doses less than $151 J cm^{-2}$. The relatively rapid return of blood flow to normal at moderate light doses (e.g., $129 J cm^{-2}$) may provide the basis for the clinical observation that recurrences of cutaneous and subcutaneous malignancies in previously treated areas may be retreated with PDT without producing skin necrosis (Dougherty, 1981). Above this threshold however, the blood flow decreases rapidly with increasing light dose. This approximates to the dose-response curve for tail necrosis (cf. Figures 1 and 3) and the theshold values were similar. Recovery in blood flow therefore may be an important factor in preventing gross tail necrosis. This is supported by the evidence from individual mice treated with a dose near the ED50. There was no significant difference between the necrosed and the recovered animals when the measurements were made 10 min or 24 h after the light component of PDT. The timing and degree of the recovery of blood flow seemed to determine the risk of necrosis in individual mice.

A different aspect of vascular function was studied by Lim *et al.* (1985). They used the accumulation of i.v. injected [$^{125}$I] bovine serum albumin in guinea pig skin following PDT as a measure of vascular permeability and vasodilatation. The increase was greatest at the completion of irradiation with the values returning to control levels by 18 h. Our observations suggest that the impairment of blood flow may be more prolonged but the disparity may be due to differences in dose or to species differences.

It is interesting to compare the time course of these functional vascular changes with the histological findings of Bown *et al.* (1986) who measured the extent of necrosis occurring in the liver of rats following PDT using either haematoporphyrin derivative or a phthalocyanine and light from an Argon pumped tunable dye laser. They found that maximum necrosis was seen at 24 h while healing began to reduce the lesion size 7 days after treatment. The same group reported on the histological appearances of rat colon up to one month following PDT with chloro-aluminium sulphonated phthalocyanine and laser light. Florid granulation tissue was observed one week after treatment (Barr *et al.*, 1987). Kaye *et al.* (1987) found maximal cerebral necrosis histologically 2 days after therapy and no significant change over the next 5 days in rats following PDT with HpD and laser light.

The underlying mechanism of the vascular effects observed here remains to be established. Direct photodynamic damage to the vascular endothelial cells may be responsible for decreasing blood flow but alternative mechanisms, for example mast cell damage (Bugelski *et al.*, 1981) or damage to platelets or red blood cells producing thrombosis may also be important. Similarly, several mechanisms may contribute to the observed recovery in blood flow such as new vessel formation, recanalisation of vessels blocked by thrombus, or decrease in tissue levels of vasoactive substances released by mast cells. The nature and time course of this recovery may however be clinically important in determining normal tissue tolerance if repeated doses of PDT are given.

We would like to thank Mr R. Swindell and Dr S. Roberts for advice and assistance with the statistical analysis of the data. This work was supported by the Cancer Research Campaign (UK).

### References

BARR, H., TRALAU, C.J., MACROBERT, A.J. & 4 others (1987). Photodynamic therapy in the normal rat colon with phthalocyanine sensitisation. *Br. J. Cancer,* **56,** 111.

BERENBAUM, M.C., HALL, G.W. & HOYES, A.D. (1986). Cerebral photosensitisation by haematoporphyrin derivative. Evidence for an endothelial site of action. *Br. J. Cancer,* **53,** 81.

BOWN, S.G., TRALAU, C.J., COLERIDGE SMITH, P.D., AKDEMIR, D. & WIEMAN, T.J. (1986). Photodynamic therapy with porphyrin and phthalocyanine sensitisation: Quantitative studies in normal rat liver. *Br. J. Cancer,* **54,** 43.

BUGELSKI, P.J., PORTER, C.W. & DOUGHERTY, T.J. (1981). Autoradiographic distribution of haematoporphyrin derivative in normal and tumour tissue of the mouse. *Cancer Res.,* **41,** 4606.

CHAUDHURI, K., GOLDBLATT, P.J., KREIMER-BIRNBAUM, M., KECK, R.W. & SELMAN, S.H. (1986). Histological study of the effect of haematoporphyrin derivative photodynamic therapy on the rat jejunum. *Cancer Res.,* **46,** 2950.

CHRISTENSEN, T., MOAN, J., SANDQUIST, T. & SMEDSHAMMER, L. (1984). Multicellular spheroids as an *in vitro* model system for photoradiation therapy in the presence of Hpd. In *Porphyrin Localisation and Treatment of Tumours,* Doiron, D.R. & Gomer, C.J. (eds) p. 381. Alan R. Liss: New York.

DOUGHERTY, T.J. (1976). Energetics and efficiency of photoinactivation of murine tumour cells containing haematoporphyrin. *Cancer Res.,* **36,** 2330.

DOUGHERTY, T.J. (1981). Photoradiation therapy for cutaneous and subcutaneous malignancies. *J. Invest. Dermatol.,* **77,** 122.

GILBERT, C.W. (1969). Computer programmes for fitting Puck and probit survival curves. *Int. J. Radiat. Biol.,* **16,** 323.

KAYE, A.H. & MORSTYN, G. (1987). Photoradiation therapy causing selective tumour kill in a rat glioma model. *Neurosurgery,* **20,** 408.

KESSEL, D, THOMPSON, P., SAATIO, K. & NANTWI, K.D. (1987). Tumour localization and photosensitization by sulfonated derivatives of tetraphenylporphine. *Photochem. Photobiol.,* **45,** 787.

KETY, S.S. (1949). Measurement of regional circulation by the local clearance of radioactive sodium. *Am. Heart J.,* **38,** 321.

LIM, H.W., YOUNG, L., HAGAN, M. & GIGLI, I. (1985). Delayed phase of haematoporphyrin-induced phototoxicity: Modulation by complement, leukocytes and antihistamines. *J. Invest. Dermatol.,* **84,** 114.

MOORE, J.V. (1987). Necrosis of murine tail skin following photodynamic treatment with meso-tetra-(p-sulphophenyl) porphine (TPPS). *Photochem. Photobiol.,* **45,** 791.

MOORE, J.V., KEENE, J.P. & LAND, E.J. (1986). Dose-response relationships for photodynamic injury to murine skin. *Br. J. Radiol.,* **59,** 257.

DE RUITER, J. & VAN PUTTEN, L.M. (1975). Measurement of blood flow in the mouse tail after irradiation. *Radiat. Res.,* **61,** 427.

SELMAN, S.H., KREIMER-BIRNBAUM, M., GOLDBLATT, P.J., ANDERSON, T.S., KECK, R.W. & BRITTON, S.L. (1985). Jejunal blood flow after exposure to light in rats injected with haematoporphyrin derivative. *Cancer Res.,* **45,** 6425.

SELMAN, S.H., KREIMER-BIRNBAUM, M., KECK, R.W., MILLIGAN, A.J., GOLDBLATT, P.J. & BRITTON, S. (1985). Correlation of tumour blood flow to tumour regression after haematoporphyrin derivative (HPD) photodynamic therapy to transplantable bladder tumours. *Adv. Exp. Med. Biol.,* **193,** 97.

SIEGEL, S. (1956). *Non parametric statistics for the behavioural sciences.* McGraw-Hill Book Company: New York.

ZHOU, C., YANG, W., DING, Z. & 4 others (1985). The biological effects of photodynamic therapy on normal skin in mice – II. An electron microscopic study. *Adv. Exp. Med. Biol.,* **193,** 111.